# Determination of birth prevalence of sickle cell disease using point of care test HemotypeSC™ at Rundu Hospital, Namibia

**DOI:** 10.1186/s12887-024-04805-z

**Published:** 2024-05-10

**Authors:** Runyararo Mashingaidze Mano, Patience Kuona, Jane Masiiwa Misihairabgwi

**Affiliations:** 1 Department of Maternal & Child Health, Division of Paediatrics, School of Medicine, Faculty of Health Sciences and Veterinary Medicine, University of Namibia Hage Geingob Campus, Bach Street, Windhoek, Namibia; 2 Department of Child, Adolescent and Women’s Health, Faculty of Medicine and Health Sciences, University of Zimbabwe, Harare, Zimbabwe; 3 Department of Human, Biological and Translational Medical Sciences, School of Medicine, University of Namibia Faculty of Health Sciences & Veterinary Medicine, Windhoek, Namibia

**Keywords:** Hemotype SC™, Newborn screening, Sickle cell disease, Malaria prone region

## Abstract

**Background:**

Sickle cell disease (SCD), a noncommunicable disease, has the greatest burden in sub-Saharan Africa. The majority of children (50–90%) with SCD die before their 5th birthday, with approximately 150,000–300,000 annual SCD child deaths in Africa. In developed countries, newborn screening (NBS) has been shown to improve the survival of children with sickle cell disease, with under5 childhood mortality reduced tenfold due to interventions performed before the development of complications. Point –of-care tests have been developed for resource limited settings to expand NBS. The aim of this study was to determine the birth prevalence of sickle cell disease in Namibia using the HemoTypeSC™ point-of-care test.

**Methods:**

A cross-sectional descriptive study was carried out at Rundu Intermediate Hospital in the Kavango East Region. Two hundred and two (202) well newborns within 72 h of birth were recruited for the study from 22 February to the 23th March 2023. Descriptive statistics were used to compute the haemoglobin types of the study participants.

**Results:**

The majority of the participants (*n* = 105, 52%) were females, and (*n* = 97,48%) were males. The median age of the participants was 23 h (Q1, Q3; 11; 33),) with an age range of 2–98 h. Sickle cell trait was present in 9.4% of the screened newborns, no homozygous disease was detected, and 90.6% had Hb AA.

**Conclusions:**

This study is the first to measure HbS gene carriage at birth using HemotypeSC point-of-care testing in Namibia. There was a moderate prevalence of sickle cell traits but no SCD. This baseline study may provide the foundation for larger epidemiological surveys to map HbS gene carriage in Namibia to provide evidence for policy makers to fashion appropriate SCD newborn screening services.

## Background

Sickle cell disease (SCD), a noncommunicable disease, has the greatest burden in sub-sub-Saharan Africa and is the most frequent genetic haemoglobinopathy, with more than 300 000 children born annually, and this number is expected to increase to 400 000 by 2050 [1–3]. The majority of these children (50–90%) die before their 5th birthday, with approximately 150,000–300,000 annual SCD child deaths in Africa, which can potentially account for 5–10% of the region’s total child mortality [1, 4, 5]. Nearly 90% of SCD patients live in 3 countries: Nigeria, India and the Democratic Republic of the Congo. In these countries, nearly 2% of the population has SCD with a carrier rate and sickle cell trait ranging from 10–30% [6]. However, it has recently been found that the distribution of all haemoglobin disorders is extremely diverse within different countries, even within small geographical distances [7].

Sickle cell disease (SCD) is a genetic autosomal recessive disorder that results from the substitution of valine for glutamine at position 6 of the beta chain haemoglobin. The haemoglobin (HbSS) tetramer that results from the substitution of alpha 2 and beta S2 is poorly soluble and rigid when deoxygenated, resulting in vaso-occlusion, which in turn causes several complications [8]. Moreover, there are different forms of sickle cell disease, including HbSS, HbSC, HbS beta-thalassaemia, HbSE, and HbSD, which occur throughout sub-Saharan Africa and in small pockets in the Mediterranean region, the Middle East and the Indian subcontinent [9]. HbSC disease is restricted to parts of western and northern Africa, and HbS thalassemia is localised in parts of sub-Saharan Africa, some parts of the Middle East and the Indian subcontinent. Additionally, HbSE commonly occurs in India, Bangladesh, Myanmar and east and southeast Asia [9].

Among SCD variants, HbSS is the most common in the sub-Saharan region and is associated with severe complications in comparison with the other variants. These complications include but are not limited to priapism, pulmonary emboli, and osteonecrosis and ultimately damage every organ system, including the spleen, retinae, kidneys and liver [10].

In the sickle cell trait, the heterozygous form of HbS is the carrier state for sickle cell haemoglobin. These individuals inherit HbS from one parent and HbA from the other parent, making them heterozygous (HbAS). More than 100 million or approximately 5% of the world’s population has the sickle cell trait (SCT) [11]. Most people with sickle cell traits live asymptomatically. Despite SCT being perceived as an asymptomatic condition, several case reports and reviews have reported an increased incidence of renal medullary carcinoma among young patients with SCT aged 9 to 69 years [12–14]. Other forms of traits include HbAC and HbAE, although these are rare forms [9].

The gene for sickle haemoglobin (HbS) is a prime example of natural selection. It is generally believed that its current prevalence in many tropical populations reflects selection for the carrier form (sickle cell trait (HbAS)) through a survival advantage against death from malaria [15]. A study by Williams et al. [15] showed that HbAS had no effect on the prevalence of symptomless parasitaemia but was 50% protective against mild clinical malaria, 75% protective against admission to the hospital for malaria, and almost 90% protective against severe or complicated malaria.

Malaria remains a major public health problem in Namibia, mostly in the Kavango East and West, Ohangwena and Zambezi regions. Kavango East and West accounted for 81%, Zambezi accounted for 10% and Ohangwena accounted for 5% of the 4 regions, according to a recently published study (2023) by Katale and Gemechu [16].

Early detection of sickle cell disease or disease-related traits is imperative for long-term outcomes, as treatment can be initiated early. In developed countries, newborn screening (NBS) has been shown to improve the survival of children with sickle cell disease, with under5 childhood mortality reduced tenfold due to interventions performed before the development of complications [17].

Over the years, several techniques have been employed to diagnose and monitor SCD. High-performance liquid chromatography (HPLC) and isoelectric focusing (IEF) are the two main laboratory techniques for haemoglobinopathy screening that are currently suitable for routine use and have been used in developed countries and several studies in Africa [18]. Molecular genetic tests are considered the gold standard tests because they target the affected genes and they can distinguish different mutations [19]. These include restriction fragment length polymorphism (RFLP) partial restriction of deoxyribonucleic acid (DNA), real-time polymerase chain reaction (PCR) [20] and DNA sequencing, which is the most expensive molecular method compared to RFLP and PCR.

Because of the reagents, instrumentation, personnel and review time required for analysis, it provides the most comprehensive data for the beta-globin gene [20]. Point-of-care tests (POCTs), SCD screening methods validated in developing countries, are easier to perform and require less qualified personnel with results available within a short period of time [21]. POCTs that have recently been developed include the HemotypeSC™, Sickle SCAN™, Heme Chip, Aqueous multiphase System (AMPS), Paper-based Sickle test (microfluid assessment) and microchip-based cellulose acetate electrophoresis test (‘Gazelle) [22]. Gazelle uses Hb electrophoresis on a much smaller machine, which actually be used in the community, but unfortunately, skilled personnel are still required to carry out this process effectively [22]. Sickle cell SCAN™, a lateral flow assay that reliably identifies HbA, HbS, and HbC, is easily performed by non‐skilled personnel and is easily interpreted, rapid test at the point of care [21]. This test detected the correct presence of A, S, and C with an overall diagnostic accuracy of 99% at the bedsid [23]. However, it is relatively more expensive than other POCTs.

Heme Chip is reliable and is able to distinguish most types of sickle cell disease, including compound heterozygotes. However, it requires skilled interpretation, is web-based, and is automated, and this approach is out of reach for most resource-limited region [21]. Additionally, the aqueous multiphase system (AMPS) (density-based test to separate Hb in fluids with different densities) and paper-based sickle test (microfluid assessment) are inexpensive and require nonskilled personnel. However, interpretation may be difficult, and the latter requires a scanner for interpretation [21, 24].

HemoTypeSC™, a POCT monoclonal antibody that targets Hb A, S, and C but not Hb F, is one of the newest techniques and yields results in 10 min [25, 26]. Multiple studies have shown that HemoTypeSC™ has a sensitivity and specificity of more than 98% compared to the gold standard methods of HPLC and IEF [23, 25, 27–31]. Various studies have shown that the HemotypeSC point-of-care testing device has high sensitivity and specificity for diagnosing sickle cell disease (SCD). A study by Olatunya et al. (2021) revealed that HemotypeSC had perfect concordance with PCR and 100% accuracy in diagnosing SCD, while Nnodu et al. (2019) reported a sensitivity of 93.4% and a specificity of 99.9% for SCD [27, 32]. In addition, Okeke, 2022 further demonstrated the feasibility of using dried blood spots with HemotypeSC, with a sensitivity and specificity of 100% compared to the standard test [31]. A further study by Adegoke et al. [33] also revealed that HemotypeSC is more sensitive than alkaline cellulose acetate haemoglobin electrophoresis. These findings collectively suggest that the HemotypeSC is a reliable and accurate tool for SCD diagnosis. Although other POCT tests, such as Sickle SCAN, have been validated in some parts, Hemotype SC has been shown to be less expensive and easier to use  [34, 35].

Newborn screening for sickle cell disease has not yet been established in Namibia. Sick children tend to present to referral hospitals with SCD-related complications prior to diagnosis. The birth prevalence of sickle cell disease and sickle cell traits has not been documented in Namibia.

The aim of this study was to determine the birth prevalence of sickle cell disease and sickle cell traits using the HemotypeSC™ point-of-care test.

The POCT detects normal haemoglobin (HbAA), SCD (HbSS, HbSC), sickle cell trait (HbAS) and/ or homozygous and heterozygous for HbC (HbCC, HbAC). This study was the first in Namibia to carry out NBS for sickle cell disease and the first to use the point-of-care test Hemotype SC™. In addition, this study will guide policy on the need to introduce SCD and NBS in Namibia, as recommended by the World Health Organisation (WHO) African Region strategy, which provides a set of public health interventions to reduce the burden of SCD in Africa. This strategy focuses on improved awareness, disease prevention and early detection [36].

## Methods

### Design and study setting

A descriptive cross-sectional study was conducted between February and March 2023 at the Rundu Intermediate Hospital postnatal ward in the Kavango East Region, Rundu, Namibia. Rundu Intermediate Hospital is the regional referral hospital for the Kavango region and caters to approximately 237,779 people according to the 2016 Inter-Census Demographic Survey (NIDS) [37]. On average, 500 to 800 babies are born at Rundu Intermediate Hospital each month. Rundu is in the northern part of Namibia, an area with a high malaria burden, making it an area of interest for sickle cell disease and/or traits. Additionally, it is close to the Angolan border, a country with a high SCD close to 2% plus a high sickle cell trait of up to 21% in the sickle cell disease belt, and migrants cross from one country to the other with possible intermarriage [34]. According to the 2013 Namibia Demographic Health Survey, 26.6% of women in the Kavango region delivered at home, and only 72.8% delivered in health facilities [38].

### Characteristics of participants

**Fig. 1:**
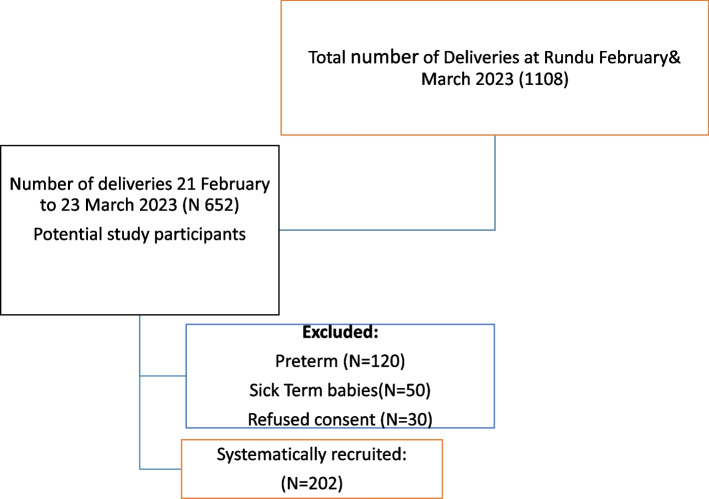
Flow diagram showing patient flow in February and March 2023 at Rundu Hospital

The study participants were term newborn babies whose mothers provided written consent to participate in the study within 72 h of birth and who were delivered at Rundu Intermediate Hospital during the study period. All well-term newborn babies who delivered at Rundu Intermediate Hospital were included in the study (see Flow diagram; Fig. 1 below). Term babies who were ill or had congenital malformations were excluded from the study.

### Sample size determination

The sample size for this study was 201. This was determined using the Dobson formula [39, 40] below as follows:

$$n={\left(\frac{{Z}_{\alpha /2}}{d}\right)}^{2}p\left(1-p\right)$$

${Z}_{\alpha /2}$ is the standard normal value corresponding to the desired level of confidence (95%).

d is the maximum allowable error, (0.05) or 5% (width of the confidence intervals).

p is the estimated prevalence of sickle cell traits or sickle cell disease in newborn babies. The calculated sample size was 201 participants based on a study in Zambia [41] in which the prevalence (p) of the sickle cell trait was 15.5%. The precision for the sample size was 5%.

### Sampling method

The study site was selected using purposive sampling because Rundu is a malaria-prone region that provides a selective advantage to sickle cell traits. The selection of participants was based on a simple random sampling approach to ensure the representativeness of the sample. We recruited every 4th newborn delivered. Health education on SCD and SCT genetics, long-term complications and the benefits of newborn screening was provided to the mothers prior to recruitment in the study. The researcher and two other research assistants performed the HemotypeSC tests. Training was carried out initially using video materials provided by the supplier and then later using actual patients. Ongoing online training continued throughout the study.

### Haemoglobin determination with the HemotypeSC™ point-of-care test

The mothers of all well-term newborn babies whose mothers gave consent within 72 h of birth were screened for sickle cell disease and sickle cell traits using the HemotypeSC™ point-of-care test (Silver Lake Research, Azusa, CA, USA). Researchers placed approximately 6 drops of water in a plain test tube prior to the point-of-care test. Approximately 1 µl of blood was collected from the heel prick onto a blood collecting strip for HemoType SC™. The blood collection strips were then placed in test tubes, and the tubes were swirled until a pink colour was obtained. A test strip for haemoglobin was then placed in the test tube and left on the bench for 10 min before reading.

After 10 minutes the haemoglobin test strip was removed from the tube and read according to the manufacturer’s instructions. The absence of a line represented the type of haemoglobin the participant had (full description of the step by step HemotypeSC™ is described in previous studies) [28, 42]. The results were interpreted as HbAA, HbAS, HbAC, HbSS, HbSC, HbCC, or invalid. HbAA indicates normal haemoglobin, HbAS indicates sickle cell trait, HbAC indicates HbC trait and HbSS, and HbSC indicates sickle cell disease. The results were entered into each infant’s questionnaire at the time the results were interpreted. A photo of each test strip, including the study ID of the infant, was taken as a backup. The Hb results were communicated immediately, and those with abnormal results had contacts collected and referred to the Paediatric Outpatient Clinic for further follow-up by the resident paediatrician.

### Statistical analysis

The data were anonymized before being analysed with the Statistical Package for Social Sciences (SPSS) version 29. Descriptive statistics were used to describe sociodemographic data as well as haemoglobin phenotypes. We calculated the proportions of newborn babies with sickle cell disease (HbSS) or HbSC, sickle cell trait HbAS or HbAC ( HbC trait), and normal haemoglobin (HbAA) levels.

## Results

The results of this study provide insight into the birth prevalence of sickle cell disease and sickle cell traits in newborns from Namibia.

### Demographic characteristics of the study participants

Two hundred and two participants were recruited into the study between 21 February and 23 March 2023 on Sundays through Fridays. Of all the participants, 105 (52%) were females and 97 (48%) were males (Table 1). The median age of the participants was 23 h (interquartile range (IQR), Q1, Q3; 11; 33 h), with an age range of 2–98 h. The mothers had a median of 3 children (Q1, Q3; 2; 4 children), with the number ranging from 1 to 8 children. All the babies included were term, with gestational ages ranging from 37–42 weeks and a median gestational age of 38 weeks, IQR (Q1, Q3; 37; 40 weeks). The lightest participant weighed 2.0 kg, with the heaviest weighing 4.63 kg. Three (1.5%) of the babies were macrosomic, with birth weights of at least 4.0 kg, while 10.4% had low birth weights less than 2.5 kg). In terms of birth length, the median IQR was 50 cm (Q1, Q3; 49; 52), with the shortest participant being 41 cm and the tallest being 58 cm. The head circumference ranged from 34–39 cm, with a median head circumference of 34 cm and an IQR of 34 cm (Q1, Q3, 34; 35 cm). The majority of the children were not exposed to HIV (982.2%). In addition, the majority of the caregivers were relatively well educated, with 80.2% having at least secondary education.

**Table 1:** Demographic characteristics of the study participants

**Variable**	**Frequency (*n*** **= 202)**	**Percentage (%)**
**Sex**		
Male	97	48
Female	105	52
**Maternal level of Education**		
None	5	2.5
Primary	35	17.3
Secondary	155	76.7
Tertiary	7	3.5
**Socioeconomic status**^b^		
Low	192	95
Middle	10	5
**Region of Origin**		
Kavango East	198	98.0
Kavango West	1	1.0
Ohangwena	1	0.5
Other	1^a^	0.5
**Mother’s HIV status**		
Negative	166	82.2
Positive	31	15.3
Unknown	5	2.5

^a^Other: Angola

^b^Low socioeconomic status = unemployed, Middle = employed

### Haemoglobin type of the study participants

**Fig. 2:**
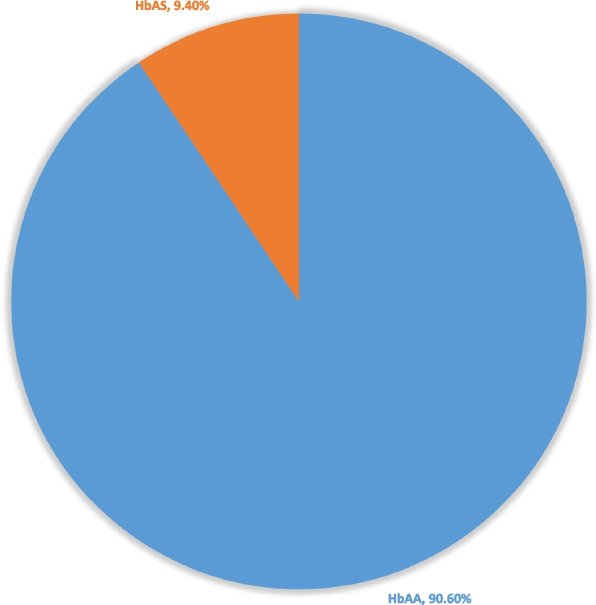
Birth prevalence of haemoglobinopathies in 202 neonates screened from 21 February to 23 March 2023 at Rundu Intermediate Hospital, Kavango region, Namibia

The distribution of haemoglobin types identified by HemotypeSC™ is shown in Fig. 2. There was no homozygous or heterozygous HbSS or HbSC. Additionally, there was no homozygous or heterozygous HbC disease,(HbCC or HbAC).

### Estimated number of people likely to have sickle cell disease in the Kavango region

The population that is likely to have sickle cell disease (HbSS) can be estimated using the Hardy‒-Weinberg equilibrium, mode, theorem or law, which states that the allele and genotype frequencies in a population will remain constant from generation to generation in the absence of the evolutionary influences such as genetic drift, natural selection and many other factors [43, 44].

For this study, the equation was used for estimation but not absolute calculation of the recessive gene.

The simple formula is p^2^ + 2pq + q^2^ = 1 and p + q = 1, where:

p = frequency of the dominant allele in the population

q = frequency of the recessive allele in the population

p^2^ = percentage of homozygous dominant individuals

q^2^ = percentage of homozygous recessive individuals

2pq = percentage of heterozygous individuals

To calculate the value of q, since heterozygous individuals (AS) represent 2pq, which is 9.4% in the study, the following equation can be set up:

2pq=0.094

Given that p+q=1, q can be calculated by rearranging the equation:

q=0.094/2p

Since p+q=1,

p=1-q,

Substituting the value of q,

p=1-0.047

=0.953

### Punnet square frequencies table

**Table:** 

	0.953(p)	0.047(q)
0.953(p)	0.908(p^2^)*	0.045(pq)*
0.047(q)	0.045(pq)*	0.002(q^2^)*

*The figures rounded to 3 decimal places.

The estimate of homozygous recessive individuals with SS for sickle cell disease, q^2^, = (0.047)^2^= 0.002209. Using this formula, and the population size of 237,779 for the Kavango region [37] given in the last census, it can be estimated that approximately 525.05 people are likely to have been born with sickle cell disease in the Kavango region (0.002209 × 237,779). The estimated figure may not be as accurate because a larger population size provides more accurate results for the equation.

## Discussion

The birth prevalence of sickle cell disease or sickle cell traits varies across different countries in sub-Saharan Africa. Our study is the first in Namibia to determine the birth prevalence of sickle cell disease and sickle cell traits. A moderate incidence of sickle cell trait birth (9.4%) was reported. However, no participant had sickle cell disease. Similar findings on HbAS sickle trait carriage were reported in Malawi, with a prevalence ranging from 6.5% to 9% % in the studies [45–47].

These findings are similar to those of a study by Munyanganizi in Rwanda, in which 987 participants did not have sickle cell disease [48]. In a study in South Africa that focused on 3 countries, South Africa, Zambia and Zimbabwe, the sickle cell trait prevalence rates were 0%, 6.5% and 12%, % respectively. This study revealed a higher prevalence of SCT compared to a study in Mozambique, which reported a prevalence of 4% [49]. Despite Rundu being closer to the Angolan border, the prevalence of SCT is twice lower than that of Angola ( prevalence 21%) [34]. These differences may be due to the different ethnic groups and, genetic makeup of the participants. In addition, a modeling study by Piel et al. 2021 reported this diversity in the distribution of the HbS gene [1].

Similar studies that used HemoTypeSC as the primary screening method in some parts of Africa reported sickle cell disease incidences ranging from 1.1 to 3.9%, with a prevalence of sickle cell traits ranging from 20.6 to as high as 31.6% [27, 50, 51]. In these studies, laboratory-based confirmatory tests were performed, and HemoType SC, a point-of-care test, was highly sensitive.

Studies that validated the accuracy of HemoTypeSC reported a sensitivity of 94.4 to 100% and specificity of 99.9 to 100% in multicentre studies conducted in Ghana, Martinique and the USA and a separate study in Nigeria [25, 27]. Therefore, our study findings may be a true representation of the actual haemoglobin types found in the participants considering the high sensitivity of the test. Considering that Namibia is a vast country with centralized laboratories which are from health facilities and limited laboratory personnel, the HemotypeSC is a potential tool for newborn screening for sickle cell disease also a cheap test for diagnosing SCD in symptomatic patients; children and adults alike. Health personnel can be trained at low-level facilities and perform the test and provide results at the one visit avoiding loss to follow up associated with centralized laboratory testing. The current test for diagnosing symptomatic patients, haemoglobin electrophoresis, has a turnaround time of between 7 to 14 days, requires highly skilled personnel and is much more expensive, costing between US$ 90 to US$200, which is 19–100 times more expensive than HemotypeSC, which costs between US$ 2 and US$5 [28].

The main issue remaining is whether or not validation is required for the Namibia population. This study was carried out with the background of the test having been validated in several studies showing high sensitivity and specificity [27, 31–33]. Perhaps the absence of detection of homozygous HbSS may raise the need for confirmatory tests in future studies prior to the introduction of NBS in Namibia.

### Limitations of the study

The limitation of this study is that, the study was only carried out at one facility, yet the Kavango region has two district hospitals and some health centres that provide maternity care. In addition, participants with sickle cell disease might have been missed because they were born outside the health facility. Additionally, patients excluded from the study could have been carriers or have sickle cell disease. Therefore selection bias could have contributed to the results of the study. Future studies should therefore expand the facilities and consider community- based NBS and include all newborn babies regardless of gestational age to obtain an accurate representative birth prevalence of SCD and SCT in Namibia. Finally, our study reported on the level of maternal education which in some countries and cultures, greater education sometimes correlates with better information about genetics of sickle cell disease so that women avoid a mate with sickle trait or HbC trait and therefore skew their offspring toward fewer births with sickle cell disease [52–54]. However we did not correlate level of education and uptake of newborn screening. Future studies could consider level of education, knowledge on SCD genetics and choice of mate.

## Conclusion

In conclusion the research serves as a foundation for future efforts to combat sickle cell disease in Namibia. There was a moderate prevalence of sickle cell trait and the estimated sickle cell disease birth prevalence. It underscores the importance of early screening accurate diagnostic tools which are affordable and easy to use. The HemoTypeSC is an inexpensive test, accurate, and rapid point‐of‐care test that can be used in resource‐limited regions to provide timely diagnosis and support newborn screening programs. Compared to higher income countries offering universal NBS screening for SCD, some questions, such as like ‘Should Namibia consider investing in universal newborn screening or targeted NBS for SCD? NBS is a key standard of care for SCD, as it enables early identification of SCD to allow early access to disease- modifying treatments, infection prophylaxis, and augmented vaccinations to minimise complications that can cause serious disability/reduced reduce quality of life and /or premature death.

## Data Availability

The data sets which support the findings of this study have been deposited  in Zenodo and can be accessed openly via 10.5281/zenodo.826621.

## Abbreviations

DBS: Dried blood spots
DNA: Deoxyribonucleic acid
Hb: Haemoglobin
HbAA: Haemoglobin AA
HbCC: Haemoglobin CC
HbS: Haemoglobin S
HbSS: Haemoglobin SS
HPLC: High-performance liquid chromatography
IEF: Isoelectric focusing
NBS: Newborn screening
NDHS: Namibia Demographic Health Survey
NIDS: Namibia Intercensus Demographic Survey
PCR: Polymerase Chain Reaction
POCT: Point of care test
REDCap: Research Electronic Data Capture
RFLP: Restriction Fragment Length Polymorphism
SCD: Sickle cell disease
SPSS: Statistical Package for Social Sciences
WHO: World Health Organisation
